# Insight into the Microbiota of Orthopteran in Relation to Gut Compartmentalisation

**DOI:** 10.3390/insects16060555

**Published:** 2025-05-24

**Authors:** Thierry Hance, Alisa Hamidovic, Siripuk Suraporn

**Affiliations:** 1 Biodiversity Research Centre, Earth and Life Institute, UCLouvain, Croix du Sud 4-5, 1348 Ottignies-Louvain-la-Neuve, Belgium; thierry.hance@uclouvain.be (T.H.); alisa.hamidovic@uclouvain.be (A.H.); 2 Department of Biology, Faculty of Science, Mahasarakham University, Kantarawichai District, Maha Sarakham 44150, Thailand

**Keywords:** Orthoptera, Ensifera, Caelifera, gut microbiota role, ecological symbioses, endosymbionts, gut compartmentalization

## Abstract

This review first provides an overview of the functional diversity of Orthoptera-associated microbiota and the services they provide to their hosts. However, data are widely scattered across the different families studied, making it difficult to establish whether a core microbiota is present. The abundance of some genera (*Pantoea*, *Enterococcus*, *Enterobacter*, *Acinetobacter*) is associated with the degradation of cellulose compounds, although their clear contribution remains to be determined. In addition, *P. agglomerans* may play a role in the production of aggregation pheromones in the desert locust. In terms of gut compartmentalisation, the diversity of the bacterial community in the foregut appears to be highly variable between individuals and species, whereas it is more uniform in other parts of the gut. Metabolic pathways of the gut microbiota revealed differences in amino acid metabolism between the midgut and hindgut. Bacteria in the midgut are associated with amino acid synthesis and anaerobic metabolism, whereas pathways in the hindgut may be involved in amino acid catabolism and ace-tyl-CoA-mediated processes. Further research is needed to better understand these different components of the bacterial community in digestive processes, and to identify bacterial species of particular interest in explaining species’ lifestyles or for bioconversion.

## Simple Summary

Orthoptera are a very diverse and globally distributed order of insects. They provide important services in grassland ecosystems, such as organic matter decomposition and vegetation renewal. They also include species that are important crop pests and are increasingly used in the insect farming industry for food and feed. Despite their ecological and economic importance, the functional diversity of their gut-associated microbiota has received little attention compared to other insect groups. However, unravelling the microbiological dimension of these insects is crucial to better understand the role of microorganisms in the evolutionary success of these mainly herbivorous insects and to optimize their rearing conditions with a view to industrial bioconversion processes and as alternative food resources.

## 1. Introduction

This article is intended for a fairly broad audience of entomological researchers and people interested in rearing insects for food or feed. It deals with the relationships between the gut microbiota and insects belonging to Orthoptera. It synthesizes the available information for economically important species such as grasshoppers, which have been the subject of a great number of publications and are of great interest to the scientific community. It also studies the gut microbiota of species used in human and animal food, a rapidly developing field that touches on several disciplines, from nutrition to microbiology and food safety. The study of the gut microbiota of insects is a rapidly developing field, and as there are few syntheses available for Orthoptera, this paper fills that gap.

Insects are commonly consumed in many countries, mainly in Africa and Asia, where they represent an abundant and cheap source of protein and are more readily available in the wild in tropical areas [1,2]. Insects have a significantly higher rate of bioconversion of plant foods, or even waste, into usable proteins than traditional mammal and poultry farming, and require less space, produce fewer greenhouse gases, and require less water [3]. This interest in insect farming comes from the valuable biomass they can quickly generate from organic waste, including secondary products such as insect frass for potential probiotics [4]. However, to promote insects as food and feed, harvesting from the wild is not an option due to the pressure exerted on biodiversity, and industrial rearing becomes necessary. This can be performed in controlled environment facilities using agricultural residues or food waste. While this type of practice is particularly important in the context of a circular economy, the microbiological quality and safety of the final product must be part of the acceptance elements of this alternative food. Populations in Western countries are not accustomed to eating insects and, therefore, strategies to “convince” consumers of their hygienic safety, environmental sustainability, and taste are necessary [5]. The insect production sector is maturing rapidly, but it still faces many challenges, which can only be addressed if all stakeholders cooperate closely. In practice, the insect industry is growing rapidly and is set to be worth USD 8 billion by 2030 [3]. The highest regional share market is in the Asia–Pacific, Europe, Latin America, North America, the Middle East, and Africa, respectively. Mexico has the highest number of insect species that are recognized as edible, followed by Thailand [6]. More than 2000 insect species are traditionally consumed [7]. However, from an industrial point of view, several insect species have become exemplary and are now mass-produced, such as the black soldier fly, *Hermetia illucens* [8], yellow meal worm, *Tenebrio molitor* [9], and various Orthoptera [10]. In Asian countries such as Thailand, grasshoppers are growing rapidly as a Thai food specialty and a driving force behind the grassroots economy for Thai farmers [11].

Orthoptera represents an extremely diverse group with more than 25,000 described species [12]. They are subdivided into two suborders: Caelifera, which includes grasshoppers and locusts, and Ensifera, which includes crickets. Most species are linked to open ecosystems and particularly grassland or crops, although they have colonized any kind of terrestrial environment. This highlights a great capacity for adaptation linked to a long evolutionary process. At the same time, we know that endosymbionts and the microbiota associated with insects offer the possibility of exploiting food resources that are deficient in certain nutrients, as well as new ecological niches [13]. Orthoptera also have a poor reputation with humans, being considered a dangerous pest leading to the loss of agricultural products [14]. For instance, migratory locusts are known to produce heavy damage to crop production during swarming episodes, leading to millions of dollars in damages and control devices [15]. At the same time, many species of Orthoptera are also used as food and feed and are considered a promising source of nutrients for humanity. Edible Orthopterans have long been used as human food, with the families Acrididae, Pyrgomorphidae, Tettigoniidae, and Romaleidae occupying some of the top positions in the world of entomophagy [16]. Crickets, grasshoppers, and locusts provide a valuable source of protein, carbohydrates, fatty acids, vitamins, minerals, and fibre [10,16,17,18,19]. While 100 g of crickets can provide 63 g of protein, 0.25 g of omega-3 fatty acids, and 5.0 mg of iron, the same quantity of beef counts for 25.6 g of protein, 0.009 g of omega-3 and fatty acids, and 2.4 mg of iron [20,21]. Besides their use as human food or animal feed, orthopteran lipids can even be used for cosmetic applications. Indeed, Ref. [22] have demonstrated that *Locusta migratoria* and the house cricket, *Acheta domesticus*, present a high potential for use in skin care (hand cream). Presently, over 60 cricket species are consumed in 49 countries globally, and farming crickets is developing quite fast. For instance, in Thailand only, more than 20,000 farms are rearing crickets for commercial purposes [10]. Four major species of crickets are reared on a large scale: *A. domesticus*, *Gryllus bimaculatus* Degeer, *Teleogryllus testaceus* Walker, and *Modicogryllus confirmata* Walker. In addition, the giant cricket, *Brachytrupes portentosus* Lichtenstein, is increasingly being bred for its taste, high protein content, and higher price [23]. The pest and food status of Orthoptera increases the interest in a better knowledge of their associated bacteria, as this may improve tools to regulate their populations, or at the same time, improve their potential for bioconversion. However, our knowledge of the microbiota associated with the digestive tract of orthopteran species used as food and feed, and its function, is still incomplete. For example, like all insects, Orthoptera have a compartmentalized digestive tract whose different parts have a determined role in the preparation of food, its digestion, and the absorption of nutrients [24]. We still lack information on the relationship between the bacteria present in the different areas and their respective potential. This is a key aspect since research has shown that insect-associated microorganisms can be used to optimize the conversion of organic waste into insect biomass, notably by promoting the digestion of certain foods and by eliminating certain pathogens, toxins, and other contaminants present in the resources digested by the insects. In a review of the role of the gut microbiota in insects, Refs. [25,26] showed that it plays multiple roles, particularly in detoxifying pesticides, providing a natural defence system, and resistance to toxins and pathogens. At the same time, some microorganisms can potentially be pathogens, either for the insect or for the animals and humans that consume them [27].

Thus, considering the Orthopteran role in energy cycles in ecosystems, the pest status of several species, as well as their potential for food and feed production, it is necessary to synthesize the role of their microbiome in food processing. In this context, it is useful to present the compartmentalization of the digestive tract of these species in relation to their physiological role in digestion, but also to particularities linked to embryological origin. We will then present our knowledge of the microbial community associated with digestive tracts and its possible involvement in cellulose and lignocellulose transformation. Finally, we will attempt to establish the relationship between bacterial communities and their location in the digestive tract.

## 2. Survey Methodology

The Scopus and Google Scholar search engines were used for an extensive literature search. The following keyword combinations were used (1) gut and microbiome and Orthoptera or grasshopper or cricket, (2) gut and microbiome and function and Orthoptera or grasshopper or cricket, (3) gut and microbiome and compartmentalisation and Orthoptera or grasshopper or cricket, (4) gut and microbiome and cellulose and degradation and Orthoptera or grasshoppers or crickets, (5) gut and microbiome and immunity and Orthoptera or grasshopper or crickets. The term “compartmentalisation” has also been used in its American form: “Compartmentalization”.

For these different combinations, we have eliminated redundant references as well as references concerning the consequences of the use of Orthoptera as food or feed on human or animal microbiomes. When very similar information was present in two publications, and to limit the number of references, we gave priority to the articles that were the first to mention this information, and then to those that gave the explanation best supported by new experimental data. We have also favoured experimental articles over review articles, except where the latter provide an original synthesis and new hypotheses. Finally, this review does not cover endosymbionts present in compartments of the insect body other than the digestive tract, nor viruses, fungi, or other non-bacterial symbionts. Moreover, considering the number of citations obtained, we decided not to make an extensive review of all descriptive papers, but to focus on publications dealing with the functional aspect and considering the compartmentalization of the digestive tract. Our aim was to summarize the main results in a pedagogical way, not to report all the research performed. Thus, our search was completed by an analysis of the older references currently cited by the various authors of the collected articles. The idea is not to present a systematic and complete review of the composition of the microbiota in Orthoptera, but to highlight the state of our knowledge on the structure-function relationships of the more global units of digestive efficiency in these species. Finally, we practiced gut dissection on several species (at least three individuals per species) to illustrate the diversity of structures in the main species used as food or feed. An Olympus/SZX16 (Tokyo, Japan) binocular loupe with 2.5× magnification was used for these dissections.

## 3. Results

**Figure 1:**
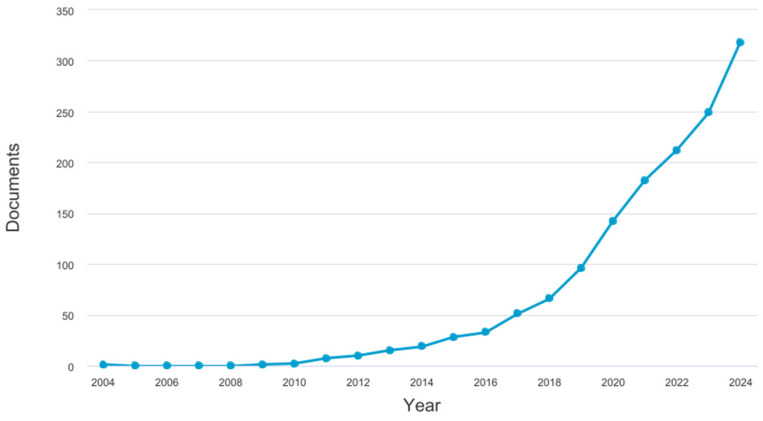
Increase in the number of publications corresponding to the keywords ‘gut and microbiome and Orthoptera or grasshopper or cricket’ according to Scopus search carried out on 21 February 2025. By 2025, 48 new articles had already been published on the subject, using the same keywords.

The number of publications concerning the gut microbiome in Orthoptera has risen sharply, as shown in Figure 1, but remained below 50 articles per year until 2017. The increase in interest from the scientific community is probably linked to the growing interest in insects in animal feed and human food, as well as to the development of metagenomic techniques and the reduction in analysis costs. A total of 93 articles, books or book chapters, and one master’s thesis were considered in this review.

### 3.1. Composition and Role of Gut Microbiome

Even if their number had increased during previous years, publications on the analysis of the microbiome of Orthoptera remain scattered. They concern different species, belonging to very different families, with different feeding habits and lifestyles. Most are based on the culture of bacteria found in the digestive tract or culturomics [28,29] and some on the V3–V4 region of the 16S rRNA sequencing [30], which often does not allow for identification further than family or genera. Two types of publications can also be distinguished: those that focus on the detailed analysis of a single species, and those that compare a number of species in order to identify lifestyle peculiarities. Four publications deal directly with a generalized description of the gut microbiota of the migratory locust *S. gregaria* linked to its pest status, plus a master’s thesis on a related South American species, *S. cancellate* [31]. For *S. gregaria*, several bacteria have been isolated and identified already in 1966, including *Escherichia coli*, *Enterobacter liquefaciens*, *Klebsiella pneumoniae*, *Enterobacter cloacae*, *P. agglomerans*, and a number of Gram-positive cocci that also appear to be present [28,32]. However, no common genus is given in these four publications on this species, although *Klebsiella* is mentioned three times. The composition of the microbiome, therefore, appears to be highly variable even at the level of the host species, influenced by whether the species is solitary or gregarious, and whether the individuals are from the field or laboratory [33,34]. We present a summary of our knowledge in Table 1.

If we consider the 16 publications in Table 1, which identify taxa down to genus level, 7 of them mention *Enterococcus*, 6 *Klebsiella*, 5 *Enterobacter*, 5 *Serratia*, and only 3 mention the genus *Weissella*. Wolbachia, a bacterium commonly found in arthropods, has only been reported twice. However, the presence of Wolbachia in crickets is well known and is associated with cytoplasmic incompatibility, but its presence in the digestive tract seems to be less common, with the bacteria more localized in the fat bodies, ovaries, and testes [47,48]. Given the diversity of studies and species explored, it is thus particularly difficult to determine if there is a real core of bacteria present in all Orthopterans phytophagous species. However, if we consider certain publications centred on related genera living in similar environments, a core of species may be found. For example, by analyzing the microbiomes of six grasshopper species sampled in the Texas coastal tallgrass prairie, ref. [30] showed that the dominant bacterial phyla overlapped between species, although there was a shift in the prevalence of one phylum over another depending on the grasshopper species. The greater differences in one species that they observed and compared to the other five studied can be explained by phylogenetic distance and different food preferences. However, they demonstrated the conservation of three phyla, Actinobacteria, Proteobacteria, and Firmicutes, in six different grasshopper species, a core common to the microbiome of many other herbivorous insects. These three phyla are regularly highlighted, but there is often a high variability in the composition of the bacterial community at the genus level [49]. In fact, the type of diet strongly influences the composition of the gut microbial community of Orthoptera. For example, by studying the microbial composition of 12 Ensifera species with carnivorous, phytophagous, or omnivorous diets, ref. [50] showed that the composition of the gut bacterial community of species with different feeding habits was significantly different, regardless of the taxonomic status of the insects sampled. In their case, Proteobacteria (45.66%), Firmicutes (34.25%), and Cyanobacteria (7.7%) were the core bacterial phyla with greater diversity highlighted in omnivorous species. The effect of diet and environment on the gut bacterial communities of the cricket, *T. oceanicus*, was tested by distinguishing between individuals caught in the field or reared in the laboratory on diets of known composition (only midgut and hindgut, ref. [51]). It was found that individuals caught in the wild had greater gut microbial diversity and a higher Firmicutes/Bacteroidetes ratio than crickets reared in the laboratory. In the same way, for *S. cancellata*, *Weissella* seems to be overrepresented in lab-reared grasshoppers compared to field-caught individuals [37]. By comparing the composition of the microbiota of *T. oceanicus* for individuals captured in the wild and as a function of diet type for individuals reared in the laboratory, ref. [50] showed that wild crickets had greater diversity and higher proportions of Firmicutes compared to Bacteroidetes. This difference may be related to a change in diet from a plant-rich diet in field populations to a more protein-rich laboratory diet based on cat food. At the same time, using descriptive metagenomic techniques, they showed that lab-reared crickets on chemically defined diets or cat food have more sequences associated with the breakdown of essential amino acids, while wild crickets have more peptidases that help release amino acids. However, the authors report that despite these differences, the community remains stable at the family level, suggesting that host genetics plays an important role [50].

In *Locusta migratoria*, the composition of the gut microbiome varies with life stage, species richness being significantly higher in eggs than in larvae and imagoes. Furthermore, in larvae, species richness was significantly higher in L1 and L2 than in L3, then decreased in fourth and fifth instar larvae and increased again in imagoes [40].

In a study of 24 species sampled in the western Swiss Alps, ref. [46] found a greater abundance of gut symbiont read counts than endosymbionts in species belonging to the Ensifera family (omnivorous cricket) than in those belonging to the Califera family (phytophagous grasshopper). The groups identified belong to the families Enterobacteriaceae, Erwiniaceae (in particular the genus *Pantoea*), Sphingomonadaceae, and Streptococcaceae. Interestingly, both types of microbiota (endo and gut symbiont) showed signs of host species specificity (greater similarity for individuals of the same species), while phylogenetically related host species did not harbour more similar microbiota than distant hosts. Finally, they showed that there was no effect of altitude on the composition of the gut symbionts of two species, *Chorthippus parallelus* and *Euthystira brachyptera*.

As [52] explains very well in her review on the importance of microorganisms in insect nutrition, there is a widespread misconception about the importance of microorganisms in the use of dietary cellulose and associated plant polymers by insects. According to her, this idea comes from our knowledge of the digestion of herbivorous mammals that do not have cellulases and depend on microorganisms for their degradation. In fact, plant biomass is largely made up of different types of cellulose and lignocellulose, which can make up as much as 50% of the content in grasses [53]. Although potentially energy-rich, these are particularly difficult compounds to digest [54], requiring a suite of cellulolytic enzymes such as endoglucanases, cellobiohydrolase, and beta-glucosidase [55,56]. The presence of these enzymes has been demonstrated in many phytophagous Orthopterans, particularly in the anterior and middle parts of the digestive tract, whereas their activity appears to be greatly reduced in the posterior part of the intestine [57,58,59]. This observation obviously does not mean that we should rule out any intervention of microorganisms in these enzymatic degradations, but that we should not expect forms of obligatory symbioses that allow this digestion. Indeed, the microorganisms that live in the digestive tracts of phytophagous species have access to large quantities of lignocellulosic material. In consequence, the digestive tract of Orthoptera also contains several species of microorganisms capable of cellulosic activity. For example, by analyzing the digestibility of cellulose and hemicellulose in different grasshopper species, ref. [54] conclude that the differences observed between species are related to the number of microorganisms that have the capacity to digest cellulose and hemicellulose at the gut level. They show a significant positive correlation with cellulose digestibility for *Pantoea* and a positive correlation with hemicellulose digestibility for *Enterococcus*, *Enterobacter*, and *Acinetobacter*. A significant positive correlation with cellulose and hemicellulose digestibility was also demonstrated by [40] in the *L. migratoria* gut with the bacteria *Weissella confusa*, *Pseudocitro bacter faecalis*, and *Lactococcus garvieae*. The same type of results was obtained by [34] based again on correlation, but in both cases, no direct evidence of cellulose digestion was provided. In another paper, ref. [60] isolated two *Bacillus* species and determined their cellulose-degrading capacity by a zone clearance assay on agar plates before identification by 16S rDNA gene sequencing. They showed that the two isolates, identified as *Bacillus safensis* strain MED1 and strain CACO, had relatively high cellulolytic activity. Using the same kind of techniques for the Short-horned grasshopper *Eyprepocnemis alacris alacris* (Orthoptera: Acrididae) collected in Tamil Nadu, India, ref. [61] identified two strains, *Klebsiella pneumoniae* and *Enterobacter xiangfangensis*, from gut suspension capable of hydrolyzing cellulose.

In the same way, ref. [41] isolated 27 lignocellulolytic bacteria strains belonging to five *Bacillus* species from the gut of the grasshoppers *Oxya chinensis*. They showed that these bacterial isolates possessed cellulolytic (100%) and ligninolytic (55.5%) activity but no xylanolytic (0%) activity. The fact that direct evidence for the involvement of the microbiome in the digestion of cellulosic compounds is difficult to demonstrate does not exclude the possibility that bacteria in the microbiome may have other functions. For instance, to our knowledge, only one team has looked at the presence of protease activity in the bacteria of the intestinal microbiota [62]. By sampling the contents of the digestive tract, followed by culturing on a primary screening solid culture medium producing a corresponding protease, 15 proteolytic bacteria were isolated from total intestinal extracts of *Gryllotalpa orientalis* harvested under natural conditions. Three bacterial strains showing high proteolytic activity were identified: *Priestia aryabhattai*, *P. megaterium*, and *S. marcescens*. This opens up new perspectives but also raises questions, as *S. marcesens* is best known for its role as a human and insect pathogen [63].

Surprisingly, ref. [32] showed that there were no significant differences between conventional and germ-free locusts in body weight, haemolymph lipid, protein, and carbohydrate contents, growth rate, development, and fecundity. They conclude that the gut microbiota does not appear to contribute significantly to locust nutrition. This result contradicts the experimental results of [64], who found a significantly higher growth rate for a normal line of *A. domesticus* compared to a germ-free line. In this last case, germ-free individuals developed more slowly and produced smaller adults with lower fecundity. On the other hand, the [32] study also provides a highly original functional analysis of the role of the gut microbiota in communication between individuals of the same species. Refs. [65,66] inoculated germ-free fecal pellets with *P. agglomerans*, a species commonly isolated from locusts. It resulted in the release of large amounts of guaiacol and small amounts of phenol, components of the locust aggregation pheromone. This production of guaiacol depends on the diet of the locusts and on vanillic acid of plant origin. However, it requires the digestion of plant matter in the locust’s digestive tract. Two other bacterial species, *K. pneumoniae* (already mentioned, see [61]) and *Enterobacter cloacae*, which is common in locust gut, also produced guaiacol from aseptic fecal pellets. Bacteria in the gastrointestinal tract may thus play an unexpected role in one of the most intriguing and economically devastating behaviours of crickets. In the same way, three antimicrobial phenolic compounds were purified from the intestines and fecal pellets of conventionally reared *S. gregaria locusts*, namely hydroxyquinone, 3,4-dihydroxybenzoic acid, and 3,5-dihydroxybenzoic acid. These three compounds were not found in the same insects when reared in a germ-free manner. The addition of *P. agglomerans* to the germ-free line restores production of antifungal compounds and protects locusts against the development of the enthomopathogenic fungus *Metarhizium anisopliae*. The authors conclude that the gut bacterial biota of the desert locust *S. gregaria* makes a substantial contribution to host defence against fungus pathogens by producing these antimicrobial compounds [32]. Also using the germ-free insect technique, ref. [67] showed in the desert grasshopper *S. gregaria* that individuals inoculated with three species of grasshopper gut bacteria and then fed an inoculum of the pathogenic bacterium *S. marcescens* showed fewer deaths than individuals without symbiotic bacteria. They thus demonstrate the role of a rich community of bacteria in the digestive tract in pathogen resistance. However, it is not clear from their experiment what the mechanism of action is, whether the symbiotic bacteria produce anti-*S. marcescens* toxins, whether there is a competition effect for nutrients, or whether the bacteria in the digestive tract improve the host’s immune response. Interestingly, we did not find any article highlighting a potential effect of microbiota composition on a better immune response of Orthoptera to their pathogens.

### 3.2. Compartmentalisation of the Digestive Tract

In a very interesting synthesis of the literature, [13] underlined the importance of the structuring of the digestive tract in the relations between the host and its microbiota. This global structure is relatively conserved in most insect species but shows variations in the relative importance of the different parts depending on the type of food ingested. Here, we make a synthesis of our knowledge at the level of Orthoptera to properly replace the importance of this compartmentalization of these relative functions.

**Figure 2:**
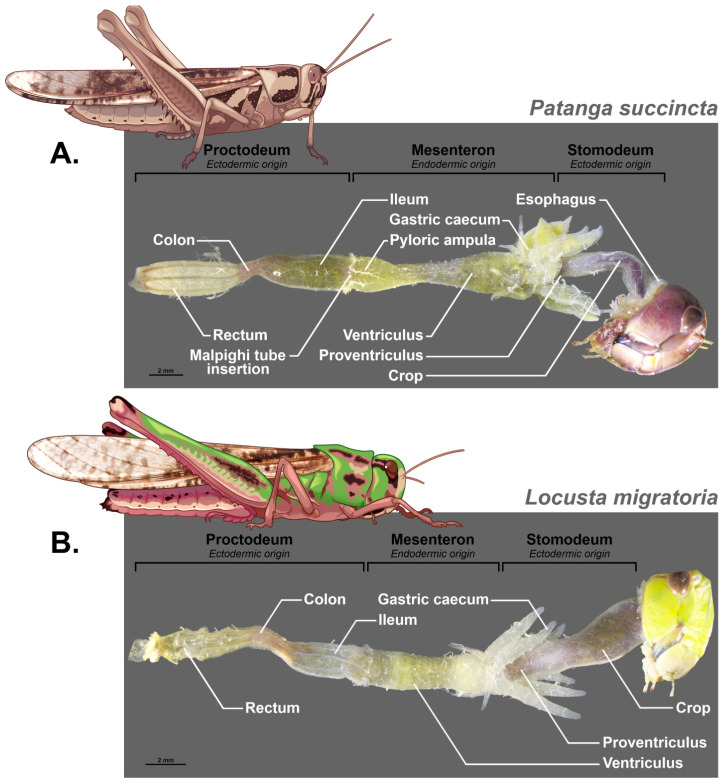
Compartmentalization of the digestive tract of Orthoptera; Caelifera based on dissection. (**A**) *Patanga succinta*, (**B**) *Locusta migratoria*.

**Figure 3:**
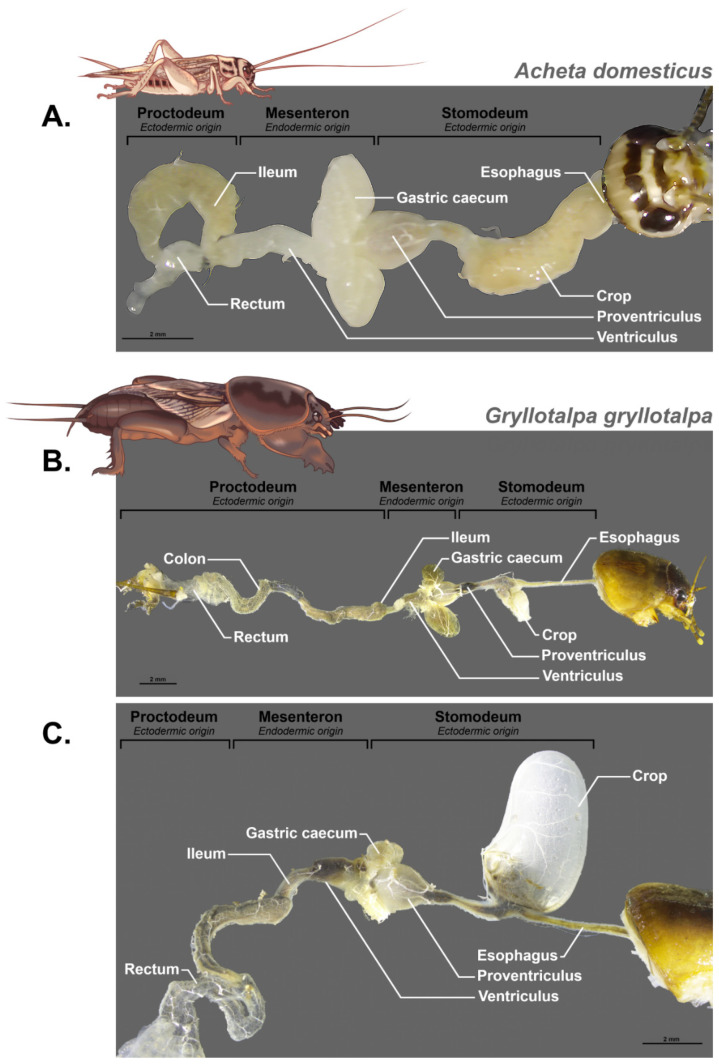
Compartmentalization of the digestive tract of Orthoptera; Ensifera based on dissection. (**A**) *Acheta domesticus*, (**B**,**C**) *Grillotalpa grillotalpa*, two different individuals, the first one with an empty crop, the second one with a full crop.

The structure of the digestive tract in Orthoptera was already described in 1939 by Hodge [68] and then by Uvarov in 1966 [69] in his synthesis “Grasshoppers and Locusts”. It was afterwards documented in different species in more detail such as in the work of [70] on the midgut of *Gampsocleis gratiosa* (Orthoptera: Tettigoniidae). Orthoptera and particularly Grasshoppers have grinding mouthparts that allow their food to be fragmented into very small pieces. The salivary ducts open anterior to the digestive tract as they join the head capsule and open into a salivary cup on the labium [71,72]. The digestive tract is made up of three distinct parts with specific embryological origins; the foregut and hindgut are ectodermal, while the midgut is endodermal (see Figure 2 and Figure 3 for some examples).

This means that during moulting at each larval stage, the anterior and posterior ectodermic parts of the digestive tract are removed. This poses a structural stability problem for the microbiota when moulting. We should therefore expect a more stable and specific microbiota at the midgut level. In addition, the pH of the lumen of each part is different and, for example, it varies from 6.1 for the foregut, 6.3 for the posterior part and 6.5 for the anterior part of the digestive caeca, it becomes basic for the midgut ventricle at 7.4 and is at 7 for the hindgut in *Abracris flavolineata* [73]. This variation in acidity must also have an impact on the microorganisms that live there.

Being of ectodermic origin, the foregut is thus covered with a layer of chitin that protects the epithelium from food bolus and which is impermeable to the passage of bacteria. This part is itself composed of a pharynx, an esophagus, and a crop whose function is to store food and is a site of carbohydrate digestion due to the activity of the salivary glands and the production of carbohydrases such as maltase and trehalase by the crop itself [73]. The foregut ends with a proventriculus that contains a sclerotized structure with spines. The role of this structure is controversial; often thought of as an internal masticatory organ, it is also sometimes considered a structure facilitating the passage of food [74]. However, in some species, this structure may be particularly elaborate with rows of teeth that suggest a role in the mastication of food [75,76]. The function of this anterior part would therefore be essentially that of preparation of the food bolus, its impregnation by salivary enzymes, and its reduction before a passage towards the midgut, also called mesenteron. This mechanical role is confirmed by the importance of the sclerotization of these proventricular structures and their morphology which depends on the type of food, phytophagous species having marked teeth and a larger proventriculus while omnivorous or predatory species have long bristles whose role would be more to guide the food towards the next compartment [76].

The embryological origin of the midgut is completely different since it is produced by endodermal cells. This means that the epithelium is not protected by a layer of cuticle and will not be affected by the moulting in the same way. However, a peritrophic membrane secreted by cells at the beginning of the midgut protects the whole and separates the food bolus in a space called the endoperitrophic space. The second phase of digestion takes place in this space, and then only small molecules can cross the peritrophic membrane to reach an ectoperitrophic space where digestion continues with the secretion of specific enzymes [77]. This peritrophic membrane has several functions. It reinforces the compartmentalization of the midgut and allows countercurrent fluid flows from the midgut that eliminate digestive enzymes and partially digested food molecules. It also ensures the protection of the epithelium against mechanical damage, plant allelochemicals, and bacterial infections [78]. It will therefore be necessary to take it into account in a study of the intestinal microbiome and its localization. However, there is no peritrophic membrane in mole crickets [79]. The midgut is composed of two parts, the ventriculus surrounded by the gastric caeca, characterized by an epithelium that shows numerous folds and several cell types [13]. The number of caeca varies within Orthoptera from two in the Ensifera (crickets and mole crickets) to six in Caelifera (grasshoppers and locusts) [80]. They can present posterior and anterior lobes [81]. A detailed description of the histology of the caeca of *Melanogryllus desertus* is given by [82] and is confirmed by the analyses of [70] on *Gampsocleis gratiosa*. They showed the numerous folds of the epithelium and their villi, indicating an absorbent but also secretory function. These folds can constitute ideal structures to accommodate bacteria [83].

Finally, the hindgut is also constituted by ectodermic cells and thus presents a cuticular protection of the epithelium. It consists of three main parts, the ileum, the colon, and the rectum, and plays an important role in the reabsorption of water and ions. Malpighian tubules are connected at the midgut–ileum junction [84].

### 3.3. Digestive Tract Compartmentation and Microbiome Function

The first study of bacterial communities in different parts of the digestive tract was carried out in 1966 by Stevenson [28] using culture techniques on the desert locust *S. gregaria*. He found a different composition of the bacterial community according to compartmentation, emphasizing that two categories of bacteria can be considered depending on the part of the gut from which they are isolated. The first group includes normal gut flora and intestinal pathogens, and the second group includes only those organisms that have accidentally entered the gut. This means that some of the bacteria present in the locust’s food or environment can be imported into the digestive tract and survive, at least in the foregut. Indeed, in a detailed analysis of the distribution of the bacterial community in the digestive tract of *T. occipitalis*, ref. [85] showed that bacterial diversity in the foregut varied greatly between individuals, whereas it was more constant in the other compartments. In the midgut, however, only bacteria adapted to the conditions of this part of the digestive tract should survive. According to the identification tools available at the time, ref. [28] identified the intestinal flora of the midgut mainly composed of *E. coli*, *Enterobacter liquefascens*, *E. cloacae*, *Klebsiella pneumoniae*, *Clostridium perfringens*, and motile streptococci. He also observed a lower diversity in the hindgut, with the presence of *E. coli* and *K. pneumoniae*, and the same species in the rectal sac with *E. liquefaciens* and *E. cloacae*. In 1981, Hunt and Charnley [86] analyzed the gut microbiome of the same species using culture techniques and light and electron microscopy. They showed a significant increase in the size of the bacterial population from the anterior to the posterior part of the digestive tract. They highlighted the presence of bacteria in the ectoperitrophic space. Interestingly, they showed the presence of *Enterobacter agglomerans* in the hindgut, now known as *P. agglomerans*. The presence of these bacteria influences energy recovery from food. Change in the abundance of the bacteria with their localization in the gut was also underlined by [42], with the highest density in the hindgut, and the lowest bacterial densities in the foregut for an endemic species of New Zealand, *Hemideina thoracica*.

Using growth and digestibility experiments and comparisons between germ-free and normal *A. domesticus* individuals, ref. [64] showed that digestive efficiency decreased in germ-free individuals. Analysis of enzyme activity from gut sections showed that the hind guts of conventional crickets had higher activity against pectin, xylan, raffinose, and locust bean gum than the mid guts or germ-free counterparts, indicating that the degradation of these polymers was of microbial origin. Using in situ hybridization studies with fluorescently labelled ribosomal probes, ref. [87] analyzed the composition of the cricket hindgut microbial community of *A. domesticus*, *Scapteriscus borelii*, *S. vicinus*, and *Gryllus rubens*. They also try to quantify the percentage of each microbial group identified. They detected Bacteroides and *Prevotella* spp, the a-, g-, and d-subgroups of the Proteobacteria, and a high G1C-content Gram-positive bacterium in the hindgut of all species examined with group-specific probes. They also found Proteobacteria b-subgroup bacteria in all species except *S. vicinus*. Finally, *Pseudomonas* spp. was presented in *A. domesticus* and *Gryllus* spp. but not in mole crickets. However, about 60% of the hindgut bacteria could not be identified with these probes. More importantly, they showed that differences in diet altered the hindgut community and abundances.

In an article devoted to the grasshopper *Zonocerus variegatus*, ref. [38] studied the microbiological composition of the different compartments of the digestive tract in the larval stages and in the adult using the plate technique, which allows live microorganisms to be counted. A fairly basic identification was carried out based on morphological aspects of the colonies. An interesting point is their identification of yeast and mould, a group that has been little studied since. They show variations in abundance between locations and stages, and the near absence of mould in adults in the midgut and hindgut. Species richness and diversity indices were analyzed to characterize gut microbial communities as a function of digestive tract compartmentation and sex in the cricket *T. occipitalis* using individuals collected in the wild and reared in the laboratory [85]. Significant differences between gut compartments were observed. In both males and females, Enterococcaceae and Budviciaceae were significantly more abundant in the midgut than in the hindgut, while Weeksellaceae was significantly more abundant in the hindgut than in the midgut. Diversity indices in females were highest in the hindgut, followed by the midgut and the foregut. Differences were also observed between sexes, indicating that the foregut and hindgut of males are associated with more diverse microbial communities than those of females, Enterobacteriaceae being, however, more frequently detected in females than in males. Enriched gut bacterial communities with high glycolytic activity were also found in the female midgut. Metabolic pathway analysis of *T. occipitalis* gut microbiota revealed marked differences between the midgut and hindgut in amino acid turnover and metabolism. The midgut shows a significant enrichment of pathways related to amino acid synthesis and anaerobic metabolism in both sexes, whereas in the hindgut, pathways may be involved in amino acid catabolism and acetyl-CoA-mediated processes. For example, amino acid (L-methionine, L-alanine) and coenzyme biosynthesis pathways were abundant in the cricket midgut, suggesting that midgut bacteria can supplement the amino acid and vitamin supply. Thus, in a contrasting way, the midgut microbiota was associated with amino acid biosynthesis pathways, whereas amino acid degradation pathways were abundant in the hindgut, highlighting the opposing roles of the bacterial community in these two parts.

As far as we know, only one other study tried to link the microbiome community, digestive tract structure, and function. It concerns the Mormon cricket *Anabrus simplex*, a species of great economic importance in North America [33]. The authors distinguished the three parts of the digestive tract, foregut, midgut, and hindgut, and highlighted a different community of bacteria characterized by a different role. The greatest bacterial richness and diversity are found in the midgut, but with a lower abundance, while the other two parts have equivalent metrics. In the foregut, *Lactobacillus* sp. is most abundant, then another lactic acid bacterium *Pediococcus* sp. is associated with the midgut, and, finally, *P. agglomerans*, an enteric bacterium, has been found in association with the hindgut. The latter is relatively more abundant in the rectum, while the composition of the ileum, which is separated from the rectum by the colon, closely resembles that of the midgut. Using PICRUSt to make predictions about metabolic capacity, they suggest that these intestinal bacteria may be involved in the metabolism of complex carbohydrates and in the defence against microbial pathogens, particularly the enteric bacteria of the midgut and hindgut and, to a lesser extent, lactic acid bacteria. On the other hand, phylogenetic analysis shows that Mormon cricket gut bacteria are closely related to bacteria associated with plants or the guts of other animals, suggesting that they can be acquired from the environment in each generation. This detailed analysis provides essential elements for our understanding of the relationships between the anatomical structure and function of symbiotic bacteria. Much remains to be performed to link the compartmentalization of the digestive tract and the functional aspect provided by the microbiota in Orthoptera, in general, and in food species in particular. Precise localization techniques using confocal microscopy and FISH would be necessary to better understand the role of certain histological structures, such as the folds of the caeca, in this complex relationship.

### 3.4. Microbiome and Food Safety

The use of insects in animal and human food obviously implies that microbiological safety elements must be taken into account [43]. It is not only during their development that insects can come into contact with potentially pathogenic organisms, but also during storage, distribution, and preparation. An analysis of the microbiota of ready-to-eat *A. domesticus* from four European countries revealed a large diversity of bacteria that differed between countries, including *Clostridium*, *Staphylococcus*, and *Lysobacter* species. The study of [43] also showed a high diversity of bacteria using whole individuals of *A. domesticus* and *G. assimilis*, thus considering also surface bacteria. However, no association was found with the actual bacteria present in the digestive tract of this species when alive or with potential sources of contamination [88].

The development of industrial breeding of insects and their packaging for sale could be a potential source of pathogenic bacteria for humans and animals [89]. Knowledge of the gut microbiome is essential to ensure the food safety of these insects. For example, ref. [90] analyzed the bacterial community present in two species of crickets, *A. domesticus* and *Brachytrupes*, not only in freshly collected individuals, but also after processing and storage. They showed the presence of enterobacteria and spore-forming bacteria, the origin of which could be contamination of live crickets by contact with soil. However, food preparation can be a key element in avoiding contamination. An analysis of the total microbial load present in whole individuals of mealworms and grasshoppers purchased from a store specializing in the sale of insects for human consumption was performed by [91] using plate counts and pyrosequencing. They showed a high load of Enterobacteriaceae, lactic acid bacteria, yeasts and moulds, and bacterial endospores. The bacterial composition differed between the two insect species, with predominantly OTUs corresponding to the genera *Weissella*, *Lactococcus*, and *Yersinia/Rahnella* in grasshoppers. They highlighted the dietary risk this poses and stressed the need for a hygienic approach to insect rearing, but also the importance of preparing these insects before consumption by appropriate heat treatment or other form of decontamination. For example, enterobacteria are killed when all insect species are boiled, provided that the cooking is long enough, at least 10 min. These bacteria are not completely destroyed when insects are simply roasted. Individuals to be roasted should therefore first be placed in boiling water for a few minutes [90]. In a food safety analysis of *A. domesticus* locusts reared in the laboratory for three generations, ref. [92] found no foodborne pathogens such as *B. cereus*, *Clostridium perfringens*, *Salmonella*, or *Listeria monocytogenes*. Rinsing of whole individuals before grinding and analysis did not reduce the microbial load, suggesting that the gut microbial load was more important than the external microbiota. However, the authors stressed that the high microbial load they observed corresponds to a high risk of spoilage and that thermal treatment should be required. In regions of Africa where insects are commonly consumed, different processing methods can reduce the potential pathogen load. These include steaming, boiling, roasting, grilling, frying, smoking, and drying, or a combination of these methods. However, this also results in a change in the food quality of the processed insects [93].

Most of these results are based on whole insect analyses. The analysis of the gut microbiome itself reveals only a part of the potential contamination, as many bacteria are present at the level of the integument as a result of their contact with the substrate. Nevertheless, this approach remains essential because it can highlight microbial species that are potentially present as intestinal symbionts and that could pose a problem to humans who consume them.

## 4. Conclusions

In recent years, a wealth of information on the Orthopteran microbiome has emerged, including the first data on the functional relationship between the structure and compartmentalisation of the digestive tract, its function, and the presence of particular clades. Our knowledge of the list of families and, in some cases, the species potentially present is growing, allowing comparisons to be made according to diet and lifestyle. The studies by [33,85] are exemplary and open a new avenue in our understanding of symbiotic relationships in the digestive tracts of these insects. However, the information available on the relationship between the digestive capacity of different substrates and the presence of specific bacteria in particular areas of the digestive tract remains fragmentary and sparse. Obviously, Orthoptera show a wide variety of diets, which is reflected in morphological and physiological differences in the compartmentalization of the digestive tract. Digestion and absorption of nutrients take place mainly in the midgut, and in this respect, the structure of the caeca is striking when we look at the diversity of folds and crypts they may contain. We already know that certain bacteria are involved in breaking down carbohydrate chains, but they also help digest cellulosic products and provide vitamins and perhaps certain amino acids. The role of the hindgut appears to be quite different, and the degradation of amino acids appears to occur as a source of energy. In the future, we can expect to see evidence of bacteria specific to these regions and probably involved in some form of digestive mutualism with their host. Finally, if it has been shown that germ-free individuals retain the ability to digest cellulose and can complete their entire development, their fitness seems affected at least in some cases. This does not exclude the possibility of specific contributions from certain molecules, such as semiochemicals, through collaboration with the microbiological community. From an agronomic point of view, the contribution of *P. agglomerans* to the production of aggregation pheromones for the desert locust, *S. gregaria*, could be better exploited for controlling this pest species [61].

In-depth knowledge of the relationships between the microbiome and Orthoptera is also of interest in terms of the economic role of the latter, either in terms of their status as crop pests or their potential contribution as a source of protein for animal and human consumption.

At the present stage, we have no data to link the microbial flora of the digestive tract of Orthopteran species to the efficiency of food bioconversion. We do have some data on the presence of bacteria that could improve the digestion of cellulosic products, but we do not know if there is any link between the microbial flora of the digestive tract and the efficiency of bio-conversion. Indeed, the study that showed the presence of these bacteria with cellulolytic properties does not allow us to certify that these bacteria play a mutualistic role for the host by improving the degradation of these compounds, which are particularly complex to digest. Finally, differences in food substrate can lead to changes in the range of microorganisms present in different parts of the digestive tract. What remains to be determined is the origin of these modifications and whether they can be translated into an improvement in the digestion process. In this context, there is a lack of data on the origin of this microbiota and on the possibility of inter-generational transmission or acquisition from the environment. It remains an extremely important task to going on in analyzing the relationship between the intestinal microbiota, the type of diet, the compartmentalization of the digestive tract and physiological functions in Orthoptera, not only because of potential health concerns, but also to better understand the interactions between the host’s own digestive physiology and the role of the microbiota in this complex process. The development of metagenomic and culturomic tools and the new imaging techniques now available should provide a wealth of information in the near future.

## Figures and Tables

**Table 1:** Synthesis of the taxonomic composition of the microbiota associated with orthopterans.

Species	Origin	Tissue	Culture of Metabarcoding	Main Identified Phyla	Main Identified Families	Main Identified Genera/Species	Reference
*Schistocerca gregaria * (desert locust)	Laboratory reared	Foregut Midgut Hindgut	Culture	N/A	Bacilli Streptococci	*Bacillus*, *Clostridium*, *Staphylococcus*, *Escherichia*, *Enterobacter*, *Klebsiella*	[28]
*Schistocerca gregaria*	Laboratory reared	Whole gut	DGGE analysis	N/A	N/A	*Enterococccus*, *Klebsiella*, *Serratia*	[35]
*Schistocerca* *gregaria*	Laboratory-reared and field-collected	Hindgut	Illumina MiSeq	Proteobacteria Firmicutes	N/A	*Enterobacter*, *Weissella*, *Spiroplasma*, *Enterococcus*	[36]
*Schistocerca gregaria* (gregarious and solitary phase)	Laboratory reared	Feces and integument	Illumina MiSeq	Proteobacteria Firmicutes Actinobacteria Bacteriodetes	Enterobacteriaceae	*Weissella*, *Klebsiella*, *Cronobacter*, *Enterobacter*, *Pantoea*, *Pediococcus*, *Enterobacillus*, *Pseudomonas*, *Pseudocitrobacter*	[34]
*Schistocerca cancellata*	Laboratory-reared and field-collected	Whole gut	16S Illumina sequencing	N/A	N/A	*Weissella*, *Methylobacterium-Methylorubrum* *Lactococcus* *Pseudomonas* *Corynebacterium* *Serratia* *Curtobacterium* *Aureimonas* *Ammorrhizobium* *Enterococcus*	[37]
*Gampsocleis gratiosa*	Bought on a market	Whole gut	Culturomics	Proteobacteria Firmicutes Actinobacteria	Enterobacterales Lactobacillales Bacillales	*Klebsiella*, *Lactococcus*, *Enterococccus* *Kluyvera*	[29]
*Zonocerus variegatus*	Field collected	Foregut Midgut Hindgut	Culture	Enterobacteriaceae	N/A	*Proteus*, *Alcaligenes*, *Escherichia*, *Serratia*, *Sreptococcus*, *Lactobacillus*, *Staphylococcus*	[38]
*Anabrus simplex* (cricket)	Laboratory-reared and field-collected	Foregut Midgut Hindgut	Illumina and Culture	N/A	Lactobacilliaceae Entorobacteriaceae Streptococcace	*Pediococcus* sp., *P. acidicatici*, *Lactobacilus* sp., *Pantoae agglomerans*, *Klebsiella* sp., *Lactococcus garvieae*	[33]
*Gampsocleis gratiosa*	Laboratory reared	Feces	Illumina MiSeq	Proteobacteria Firmicutes Thermotogae Bacteroidetes Acidobacteria Actinobacteria Chloroflexota Euryarchaeota Verrucomicrobia	Enterococcaceae Lactobacillaceae Streptococcaceae Burkholderiaceae Enterobacteriaceae Pseudomonadaceae Petrotogaceae	*Kluyvera Obesumbacterium Buttiauxella* *Lactobacillus Hafnia* *Weissella* *Lactococcus* *Burkholderia* *Citrobacter* *Escherichia* *Serratia*	[39]
*Locusta migratoria*	Laboratory reared	Whole gut	16S rRNA, Sanger sequencing	Proteobacteria Firmicutes Bacteroidetes Actinobacteria	N/A	*Enterobacter * *Weissella * *Lactococcus* *Pseudocitro bacter * *Kluyvera* *Providentia* *Serratia*	[40]
*Oxya chinensis*	Field collected	Whole gut	16S rRNA	Firmicutes	N/A	*Bacillus wiedmannii*, *Bacillus marcorestinctum*, *Bacillus halotolerans*, *Paenibacillus zanthoxyli*, *Bacillus hominis*	[41]
*Hemideina thoracica*	Field collected	Foregut Midgut Hindgut	16S rRNA	Actinobacteria Bacteroidetes Deferribacteres Firmicutes Proteobacteria Verrucomicrobia	Coriobacteriaceae Bacteroidaceae Porphyromonadaceae Rikenellaceae Deferribacteraceae Lactobacillaceae Leuconostocaceae Christensenellaceae Clostridiaceae Defluviitaleaceae Lachnospiraceae Ruminococcaceae Erysipelotrichaceae Desulfovibrionaceae Unclassified Verrucomicrobiaceae	N/A	[42]
*Conocephalus fasciatus*, *Conocephalus strictus*, *Orchelimum concinnum*, *Orchelimum vulgare*, *Paroxya atlantica*, *Scudderia texensis*	Field collected	Whole gut	Illumina MiSeq	*Proteobacteria * *Actinobacteria* *Firmicutes* *Tenericutes*	Listeriaceae Lactobacillaceae Methylobacteriaceae Rhizobiaceae Sphingomonadaceae Enterobacteriaceae Pseudomonadaceae	N/A	[30]
*Acheta* *domesticus*, *Gryllus assimilis*	Laboratory-reared and field-collected	Surface and whole body	Next-generation sequencing and Illumina MiSeq	Parabacteroides Bacteroides Firmicutes	Porphyromonadaceae Bacteriodaceae Rikenellaceae Enterobacteriaceae Verrucommicrobiaceae Pseudomonadaceae	*Lactococcus*, *Candidatus*, *Azobacteroides*, *Coprococcus*, *Enterococcus*, *Akkermansia*, *Acinetobacter*	[43]
*Acrida cinerea*, *Trilophilia annulata*, *Atractomorpha sinensis*, *Synergus mongolicus*	Field collected	Whole gut	Illumina HiSeq	Proteobacteria Firmicutes Cyanobacteria Actinobacteria Bacteroidetes Tenericutes Fusobacteria	Enterobacteriaceae Streptococcaceae Enterococcaceae Moraxellaceae Anaplasmataceae Staphylococcaceae Microbacteriaceae Clostridiaceae Xanthomonadaceae Brevibacteriaceae	*Klebsiella* *Staphylococcus* *Wolbachia* *Acinetobacter Pantoea*, *Enterococcus* *Stenotrophomonas Microbacterium Brevibacterium Corynebacterium* *Lactococcus*	[31]
*Oxya chinensis*, *Paratettix meridionalis*, *Gastrimargus marmoratus*, *Calliptamus abbreviatus*	Field collected	Whole gut	Illumina MiSeq	Firmicutes Proteobacteria	N/A	*Bacillus Enterobacter Enterococcus* *Pantoea Halomonas*	[44]
*Mecopoda niponensis*, *Ocellarnaca emeiensis*, *Gryllotalpa orientalis*	Field collected	Midgut Hindgut	Illumina MiSeq	Proteobacteria Mucoromycota Basidiomycota Firmicutes	N/A	*Intestinimonas massiliensis*, *Leclercia adecarboxylata*, *Acinetobacter baumannii*, *Klebsiella pneumoniea*, *Kluyveria criocrescens*, *Lactococcus lactis*	[45]
24 Species	Field collected	Whole gut	16S rRNA	N/A	Enterobacteriaceae Erwiniaceae Sphingomonadaceae Streptococcaceae	*Wolbachia* (super group A, B and F) *Spiroplasma* *Pantoea*	[46]

## Data Availability

No new data were created or analyzed in this study.
